# BTK blocks the inhibitory effects of MDM2 on p53 activity

**DOI:** 10.18632/oncotarget.22543

**Published:** 2017-11-20

**Authors:** Miran Rada, Mohammad Althubiti, Akang E. Ekpenyong-Akiba, Koon-Guan Lee, Kong Peng Lam, Olga Fedorova, Nickolai A. Barlev, Salvador Macip

**Affiliations:** ^1^ Department of Molecular and Cell Biology, Mechanisms of Cancer and Aging Laboratory, University of Leicester, Leicester, UK; ^2^ Department of Biochemistry, Faculty of Medicine, Umm Al-Qura University, Mecca, Saudi Arabia; ^3^ Bioprocessing Technology Institute, A*STAR, Singapore; ^4^ Institute of Cytology, RAS, Saint-Petersburg, Russia

**Keywords:** BTK, p53, MDM2, phosphorylation, ubiquitination

## Abstract

p53 is a tumour suppressor that is activated in response to various types of stress. It is regulated by a complex pattern of over 50 different post-translational modifications, including ubiquitination by the E3 ligase MDM2, which leads to its proteasomal degradation. We have previously reported that expression of Bruton’s Tyrosine Kinase (BTK) induces phosphorylation of p53 at the N-terminus, including Serine 15, and increases its protein levels and activity. The mechanisms involved in this process are not completely understood. Here, we show that BTK also increases MDM2 and is necessary for MDM2 upregulation after DNA damage, consistent with what we have shown for other p53 target genes. Moreover, we found that BTK binds to MDM2 on its PH domain and induces its phosphorylation. This suggested a negative regulation of MDM2 functions by BTK, supported by the fact BTK expression rescued the inhibitory effects of MDM2 on p53 transcriptional activity. Indeed, we observed that BTK mediated the loss of the ubiquitination activity of MDM2, a process that was dependent on the phosphorylation functions of BTK. Our data together shows that the kinase activity of BTK plays an important role in disrupting the MDM2-p53 negative feedback loop by acting at different levels, including binding to and inactivation of MDM2. This study provides a potential mechanism to explain how BTK modulates p53 functions.

## INTRODUCTION

p53 is a tetrameric transcription factor that plays a critical role in tumour suppression [1] and prevents the emergence of transformed cells [2]. In response to different stress signals, p53 mainly acts as a transcription factor to induce the expression of genes involved in apoptosis, cell cycle arrest or senescence, among other responses. Notably, p53 regulates the expression of both coding and non-coding genes [3]. p53 functions depend on many factors, including stability, intracellular distribution [4–6], interaction with other proteins [7–11] and post-translational modifications (PTMs) [7, 12–14]. Known p53 PTMs include phosphorylation, acetylation, methylation, ubiquitination, SUMOylation, neddylation, N-acetylglucosamine (O-GlcNAcylation) and poly ADP-ribosylation [5, 11, 12, 14–17]. Cell fate decisions after p53 induction can be further modulated by other factors, such as intracellular ROS levels [18, 19].

It is well accepted that ubiquitination is fundamental regulatory mechanism of many proteins, including p53. The underlying mechanism is the nuclear export and proteasome-mediated degradation of the polyubiquitinated protein [16, 20]. The most extensively studied E3 ligase for p53 is MDM2, a p53 target gene that blocks its activity, thus establishing a negative feedback loop [10, 21]. In response to DNA damage, ATM phosphorylates p53 at serine 15 (S15) and induces S20 phosphorylation by Chk2 [22]. Simultaneously, MDM2 is phosphorylated by ATM (on serine 395) [23]), ATR (on serine 407) [24] and c-Abl (near the C-terminus) [25]. The goal of this series of events is to disrupt the ubiquitination of p53 by MDM2, thus preventing protein degradation in proteasomes and allowing stabilization of the p53 protein [26, 27]. In addition, other studies have shown protein kinase B-mediated MDM2 phosphorylation at S166 and S188, which inhibits MDM2 self-ubiquitination and results in MDM2 stabilization [28–30].

Previous studies have reported phosphorylation of many residues of p53, including S15, S20, S46, S315, S373, S376, and S392 [31]. Many of these modifications mediate the induction of p53 activity or increase p53 stability through the uncoupling of p53-MDM2 [32]. Phosphorylation of p53 is exerted by a broad range of different kinases, including ATM, ATR, DNA-PK, Chk1 and Chk2 [33]. In response to stress signals, both S15 and S20 are phosphorylated, and this results in an interruption of the p53-MDM2 binding [9, 32]. Furthermore, we have shown that methyltransferase Set7/9 that methylates p53 on K372 also interacts with Mdm2 thereby sequestering it away into a non-functional complex thereby increasing acetylation and stabilization of the former [34, 35].

We previously found that Bruton’s Tyrosine Kinase (BTK) induces the phosphorylation of p53 at the N-terminus (mainly at serine 15) and that this has an important effect in its protein levels and activity [36]. BTK is a dual-specificity protein kinase able to phosphorylate both serines and tyrosines [37] that belongs to the Tec family of kinases [38]. It is an essential regulator of B cell proliferation and survival by being involved in pathways that signal for B cell development and maturation [38]. Although it has been mainly associated with B-cell malignancies [39], some studies have also suggested a pro-apoptotic and tumour suppressor role for BTK [40–44]. We showed that inhibition of BTK interfered with the induction of p53 target genes, which lead to a severe impairment in the induction of p53-mediated apoptosis and senescence [36]. This proves that BTK has an important regulatory role as an upstream component of the p53 pathway and suggests that there could be more unknown targets for its phosphorylation activity implicated in tumour suppression.

Here, we further explore the impact of BTK in the p53 pathway by investigating its effects on MDM2. We found that BTK reinforces its upregulation of p53 activity by phosphorylating and inhibiting MDM2. Our results confirm the relevance of BTK as a novel modulator of p53 activity and provide a mechanistic explanation for its regulatory role in the p53 pathway.

## RESULTS

### BTK induces the expression of MDM2

**Figure 1:**
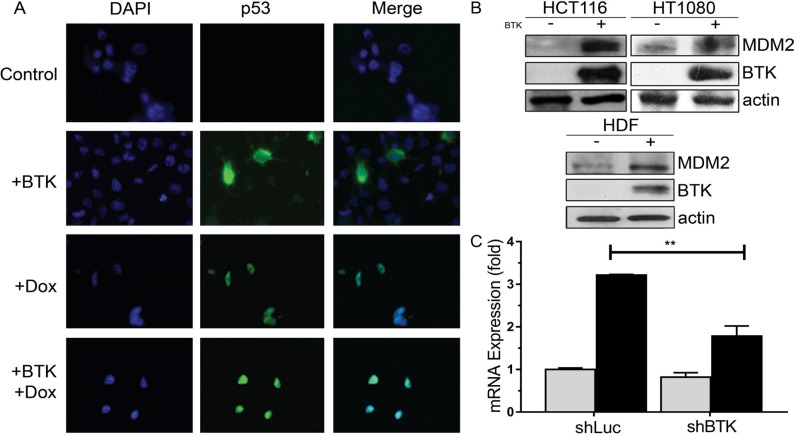
Effects of BTK on MDM2 expression **(A)** Representative immunofluorescence images of HCT116 cells transfected with an empty vector (Control) or BTK and treated with 1.5 µM doxorubicin for 20 h, showing expression of p53. DAPI is used to stain the DNA **(B)** Representative Western Blots showing MDM2 and BTK protein levels in lysates of HCT116, HT1080 and HDF transfected with BTK or an empty vector (Control) and all treated with 1.0 μM doxorubicin for 24 hours. β-actin levels are provided as loading controls, as in all other blots. **(C)** Real time PCR data showing relative expression of MDM mRNA in HCT116 stably transfected with a shRNA against luciferase (shLuc, as a control) or BTK, and treated with 1.5 µM doxorubicin for 24h. Bars represent mean values of three experiments and error bars show standard deviation. ^**^p < 0.01.

We have shown that BTK increases p53 protein levels and activity [36], which can happen in the presence or absence of DNA damage in cells with wild type p53 (Figure 1A). We observed that the expression of BTK also induced the upregulation of MDM2 protein levels in normal and cancer cells with wild type p53, even in the absence of damage (Figure 1B and Supplementary Figure 1A), consistent with the critical role of BTK in the activation of p53 target genes [36]. Indeed, we found that lack of BTK expression decreased the localization of p53 to the MDM2 promoter, as well as that of other target genes, and we also confirmed that BTK overexpression had the opposite effect, as we previously showed [36] (Supplementary Figure 1B). As expected, inhibition of BTK expression suppressed the induction of MDM2 mRNA by DNA damage (Figure 1C).

### BTK binds to and phosphorylates MDM2 to increase its levels

**Figure 2:**
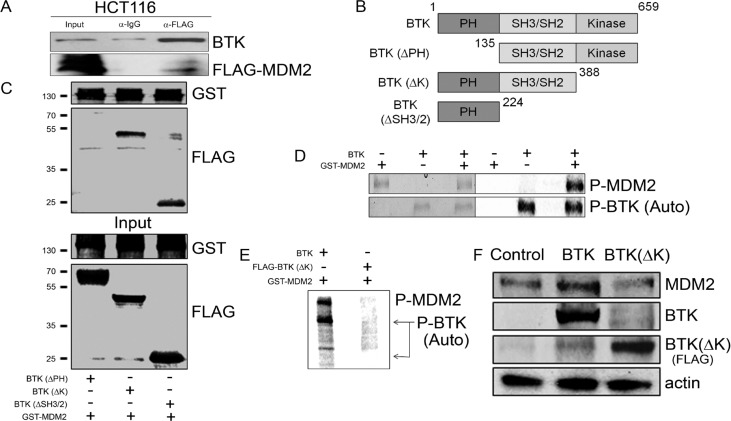
BTK binds to and phosphorylates MDM2 **(A)** Immunoprecipitation (IP) with lysates of HCT116 transfected with BTK and FLAG-tagged MDM2. IP was performed with a FLAG or IgG antibody and the Western blots used BTK and FLAG antibodies. **(B)** Schematic representation of the BTK constructs used in C. **(C)** Binding assay in HCT116 cells transfected with different BTK constructs and GST-tagged MDM2. The top blot shows binding to glutathione beads; the bottom blot shows input levels. Numbers on the left indicate approximate protein weight (kDa). **(D)** *In vitro* phosphorylation assay showing phosphorylation of MDM2 by BTK as well as BTK autophosphorylation. **(E)** Same as (D) using a wild type BTK construct or a FLAG-tagged BTK lacking the kinase domain (BTK(ΔK)). **(F)** Western blots showing MDM2 and BTK protein levels in lysates of HT1080 transfected with an empty vector (Control), BTK or FLAG-BTK(ΔK), with all samples treated with 1.5 µM doxorubicin for 24 h.

The fact that BTK disrupts the MDM2-p53 regulatory loop raised the possibility that BTK may also have a direct effect on MDM2. To test this, we first explored whether BTK could bind to MDM2. An immunoprecipitation assay indicated that this was indeed the case (Figure 2A). Using different BTK deletion constructs [45] (Figure 2B), we determined that this binding likely takes place at the PH domain (Figure 2C).Moreover, *in vitro* kinase assay showed that BTK phosphorylated MDM2 (Figure 2D). Of note, when a kinase dead BTK that could not phosphorylate MDM2 (Figure 2E) was used instead, the induction of MDM2 by BTK was lost (Figure 2F and Supplementary Figure 1C). These data together suggest that BTK binds to MDM2 and phosphorylates it, which induces an increase in MDM2 protein levels.

### BTK phosphorylation blocks the negative effects of MDM2 on p53

**Figure 3:**
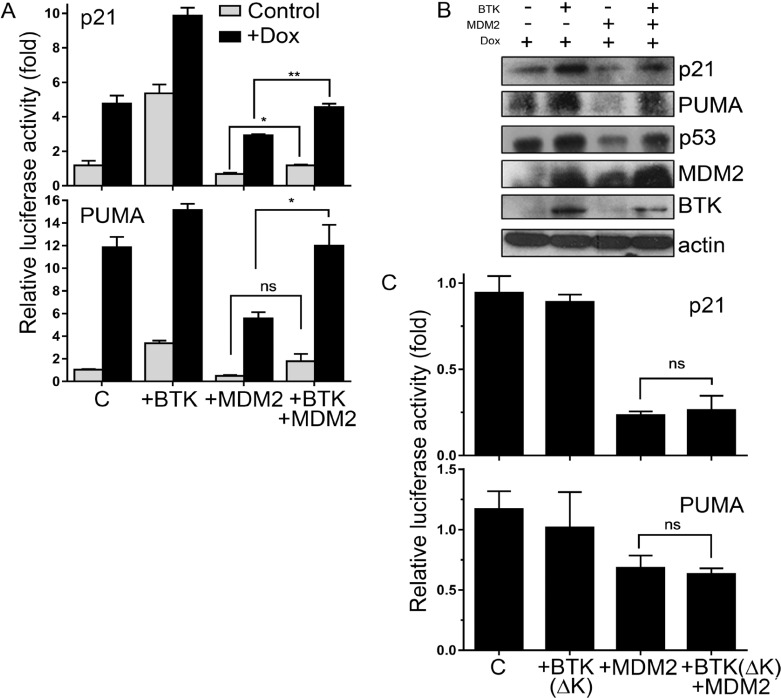
BTK prevents MDM2 inhibition of p53 transactivation functions **(A)** Relative luciferase activity of p21 and PUMA reporters in HCT116 cells in which BTK and/or MDM2 has been transfected 24 h before, in the absence or presence of 1.5 µM doxorubicin for 24 h. Control cells (C) were transfected with an empty vector. Graphs represent mean of a triplicate experiment, error bars represent standard deviation. ns: non significant; ^*^P ≤ 0.05; ^**^P ≤ 0.01. **(B)** Western blot showing protein levels of p21, PUMA, p53, MDM2 and BTK in the same cells. **(C)** Relative luciferase activity of p21 and PUMA reporters in HCT116 cells in which BTK(ΔK) and/or MDM2 have been transfected 24 h before, all in the presence of 1.5 µM doxorubicin for 24 h. Control cells (C) were transfected with an empty vector.

Phosphorylation of MDM2 is known to affect its E3 ubiquitin ligase activity [29]. Thus, we hypothesized that although the BTK-dependent phosphorylation of MDM2 increases its protein levels, it could also block its functions. Blunting Mdm2 activity would alleviate the inhibition of p53 functions by preventing its ubiquitination, which would be consistent with the effects of BTK on p53 levels and activity [36]. Indeed, the simultaneous expression of MDM2 and BTK rescued the negative effects of MDM2 on the induction of *bona fide* p53 target genes, p21 and PUMA, after DNA damage (Figure 3A and 3B). When the kinase dead BTK was used instead, this effect was not observed (Figure 3C), indicating that it depends on the ability of BTK to phosphorylate MDM2. These results prove that although BTK increases MDM2 expression, it also prevents its inhibition of p53 transactivation functions, thus resulting in upregulation of the p53 target genes.

### Phosphorylation of MDM2 by BTK suppresses its ubiquitination activity

**Figure 4:**
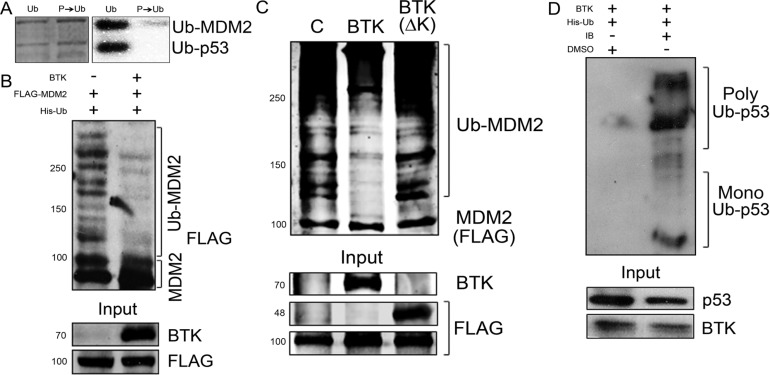
BTK inhibits MDM2 ubiquitination activity **(A)** *In vitro* ubiquitination assay of p53 and GST-tagged MDM2, before or after using BTK to phosphorylate MDM2. Left panel shows total amounts of protein loaded in the gel and right panel shows a Western blot of ubiquitinated proteins. **(B)** *In vivo* ubiquitination assay using HT1080 cells transfected with BTK and FLAG-tagged MDM2. **(C)** *In vivo* ubiquitination assay using HT1080 cells transfected with an empty vector (C), BTK or FLAG-BTK(ΔK). All samples were also transfected with FLAG-MDM2 and His-Ubiquitin. Numbers on the left indicate approximate protein weight (kDa). **(D)** Same as B, in HCT116 cells treated or not with 0.5 µM ibrutininb (IB) for 24 h, showing poly- and mono-ubiquitinated p53.

To better understand the mechanism involved in this phenomenon, we determined the effects of BTK on the ubiquitination activity of MDM2. As shown in Figure 4A, prior phosphorylation of MDM2 by BTK reduced the MDM2-mediated ubiquitination of p53 and MDM2 itself *in vitro*. Of note, presence of active BTK also suppressed auto-ubiquitination of MDM2 *in vivo* (Figure 4B), but not when a dead kinase BTK was used (Figure 4C). An affinity pulldown assay using Ni^2+^ beads confirmed that p53 was not ubiquitinated in the presence of BTK and that this was reverted when BTK activity was blocked with ibrutinib, a specific inhibitor [46, 47] (Figure 4D). Taken together, these results indicate that BTK phosphorylates MDM2 and inhibits its functions, specifically the ubiquitination of p53, which provides a mechanism to explain the increased p53 protein stability induced by BTK [36].

## DISCUSSION

Numerous regulatory mechanisms that affect p53 activity have been extensively studied for decades. Yet, novel additional modulators of p53 are still being discovered and the complexity of its network keeps increasing, which signifies the importance of p53 functions to the cell. Here, we provide a mechanistic explanation for the modulation of p53 activity by BTK, a newly described component of the p53 regulatory network. We had previously observed that BTK expression increased the stability of p53 and its transactivation abilities [36]. Conversely, blocking BTK reduced the induction of p53 after DNA damage. Consequently, cellular responses to p53 up-regulation, such as apoptosis and senescence, were greatly influenced by the expression of BTK.

We hypothesized that the role of BTK in the response of p53 to stress was determined by the ability of BTK to induce the phosphorylation of p53 at the *N* terminus and, specifically, at serine 15. It has been previously shown that the N-terminus phosphorylation serves as the initial response to damaging signals, triggering a cascade of post-transcriptional modifications necessary for p53 stabilization and activation [48]. Thus, the fact that BTK has an impact on these phosphorylation events suggests that it is a key upstream regulator of the p53 pathway.

In this study, we further elucidated the mechanisms involved in these processes. Loss of MDM2-mediated ubiquitination of p53 is widely associated with p53 phosphorylation at the *N*-terminus [5]. The interplay between phosphorylation and ubiquitination is regulated by two mechanisms: MDM2-p53 uncoupling [49] and inhibition of MDM2 E3 ubiquitin ligase activity [23]. We reasoned that BTK could have an impact on the MDM2-p53 relationship by acting on MDM2 as well. In this report, we show that this is indeed the case.

Our results prove that BTK expression increases the protein levels of MDM2 by binding to it and phosphorylating it. Although it may seem counterintuitive, this is consistent with the fact that MDM2 is a p53 target gene. Therefore, like other p53 targets, it is likely to be upregulated by the BTK-dependent activation of p53, as previously reported [36]. However, we found that this increase in MDM2 levels does not correlate with an increase in its activity. On the contrary, the ability of MDM2 to ubiquitinate p53 or itself was diminished in the presence of BTK. Our data suggest that this inhibition of the ubiquitination functions could be the result of BTK phosphorylating MDM2, although a BTK-mediated phosphorylation of p53 could have an indirect effect as well. Further experiments are needed to determine whether BTK can phosphorylate MDM2 directly, as our binding and *in vitro* phosphorylation assays would suggest, or there is an indirect mechanism involved. Also, it would be interesting to determine whether BTK may be acting in other ways to reinforce its positive effect on p53 activity.

Our results indicate BTK affects p53 activity at several levels, some of which are yet to be fully characterized. On one hand, phosphorylation of p53 at serine 15 disrupts the MDM2-p53 interaction. At the same time, phosphorylation of MDM2 by the same kinases, including BTK, should reduce its ubiquitination activity. This model underscores the important role of BTK in the facilitation of the p53 pathway and highlights how this goal can be achieved by simultaneous induction of more than one response with a similar outcomes. Moreover, the present report also stresses that the tumour suppressor functions of BTK are important and can be physiologically relevant in the context of preventing the growth of solid tumours. Since BTK inhibitors are already being used clinically to treat different leukaemias, it is urgent to better characterize the mechanisms by which BTK affects the oncogenic and tumour suppressor pathways, in order to avoid unexpected long-term side effects.

## MATERIALS AND METHODS

### Plasmid constructs

The plasmids generated and used were pGL3-p21, pCDNA3-p53, pSV-β-Gal, CMV6-, pGEX-6His-Ubiquitin, pGEX-MDM2 and pCDNA3-FLAG-MDM2, as well as BTK (OriGene RG211582). pCDNA3 was used as empty vector for controls.

### Cell culture and transient transfections.

HCT116 colon cancer cell line (with wt p53), HT1080 osteosarcoma cell line (with wt p53) and human diploid fibroblasts (HDF) were cultured in DMEM and incubated at 37°C with 5% CO_2_. After 24–48 hours, media was changed. Cells were cultured and grown until 70–80% was reached, and transfections were carried with Turbofect (Thermo Scientific, # R0531), following manufacturer’s instructions. Dilution of plasmids was performed in serum-free medium (Opti-MEM, GIBCO, Life Technologies). Cells were incubated with the plasmid/Turbofect mix for 24–48 hours and then collected. 1.5 μM doxorubicin (Sigma-Aldrich) was added to induce DNA damage. 0.5 μM Ibrutinib (Sellechem, PCI-32765) was used to inhibit BTK. 2 μM MG-132 was used to inhibit the proteasome.

### Immunoblot analysis

For lysate extraction, the medium was removed and plates were washed once with 1x PBS, trypsinized, collected and kept on ice. Cells were ruptured by passing through a syringe 5 times or with sonication, and centrifuged for 15 minutes. The supernatant was transferred into Eppendorf tubes, and protein concentrations were determined using the Bradford protein assay (Fermentas). 10–20 μg of total protein per sample were subjected to 10% or 6% SDS-PAGE and transferred to Immobilon-P membranes (Millipore). Primary antibodies used were: β-actin (Abcam, #ab8227), p53 (Calbiochem, #Op43), p21 (Santa Cruz Biotechnology, #sc-53870), PUMA (Cell Signalling, #4976), BTK (Cell Signalling, #8547S), MDM2 (Abcam, #ab137413) and Streptavidin-Biotinylated HRP Complex (GE Healthcare, # RPN1051).

### ChIP assays

ChIP assays were performed as previously described [36]. 3 × 10^6^ cells per sample were used. PCR was performed with 1 μl of DNA. The p53 (Ab-6, Galbichem#OP43) antibody was used. Primers used: 5′-mdm2 (GGTTGACTCAGCTTTTCCTCTTG), 3′-mdm2 (GGAAAATGCATGGTTTAAATAGCC), 5-Puma: (GCGAGACTGTGGCCTTGTGT), 3′-Puma (CGTTCCAGGGTCCACAAAGT), 5′-p21 (GTGGCTCTGATTGGCTTTCTG), 3′-p21 (CTGAAAACAGGCAGCCCAAG).

### Protein-protein interactions

For *in vivo* protein-protein binding assays, cells were transfected with FLAG-MDM2 and BTK, and lysed in lysis buffer (PBS, 50 mM NaCl, 1% NP-40, 1 mM PMSF) at 4°C. Proteins were immunoprecipitated with anti-IgG (Santa Cruz Biotechnology, # sc-2025), or anti-FLAG antibodies overnight at 4°C, and then protein A/G agarose beads (Millipore, #16-157) were added for 4 h with rotation at 4°C. Bound proteins were analysed by Western blotting. For *in vitro* protein-protein binding assays, cells were transfected with FLAG tagged BTK plasmid (BTK (ΔPH), BTK (ΔK), or BTK (ΔSH3/2)). The cells were lysed and BTK proteins were prepared by immunoprecipitation using anti-FLAG antibody. The purified BTK proteins were mixed with GST-MDM2 individually and incubated for 4 hours with rotation at 4°C. The mixtures were added to glutathione HiCap matrix beads (Qiagen #30900) and incubated for 4 hours at 4°C with rotation. The beads were washed with PBS and eluted, followed by Western blotting using anti-GST and anti-FLAG antibodies.

### Ni^2+-^NTA beads pulldown

Cells were transfected with BTK and 6His-Ubiquitin vectors, and after 24 hours treated with MG132 (Sigma-Aldrich, #M7449), for 12 hours. Cells were then lysed with 6M Guanidine hydrochloride (Sigma-Aldrich, #G3272), and 6His-ubiquitinated proteins were purified on Ni-NTA beads (Qiagen, #30210). The bound proteins were eluted and analysed by immunoblotting with p53-specific antibody (Calbiochem, #Op43).

### Luciferase reporter assay

100,000 cells per well were seeded in 0.5 ml growth media in 24-well plates. The following day, transfection was performed following the protocol described above. 1.0 μg total plasmid DNA (0.3 μg luciferase construct, 0.2 μg β-galactosidase construct, and 0.5 μg empty control vector or BTK construct) was used. 24 hours after transfection, media was replaced and part cells treated with 1.0 μM Doxorubicin. 24 hours later, lysates were collected in lysis buffer and stored at –80°C overnight. Prior evaluation, β-galactosidase substrate (10 ml of β-galactosidase stock solution, 20 mg o-nitrophenyl-β-d-galactopyranoside (ONPG) and 35 μl β-mercaptoethanol) was prepared and 100 μl was added to 80 μl cell lysate (per well) in a 96-well transparent plate. This was incubated at 37°C for 15 minutes. 20 μl of the cell lysate were transferred to a 96-well opaque white plate and read in a luminometer, using a luciferase kit (BioVision#K801-200), as previously described [50].

### Quantitative real-time PCR

RNA purification, cDNA preparation, and qRT-PCR were performed as previously described [51]. Primers used: GAPDH: GGGAAGGTGAAGGTCGGAGT (FWD), TTGAGGTCAATGAAGGGGTCA (REV). MDM2: TGGCGTGCCAAGCTTCTCTGT (FWD), ACCTGAGTCCGATGATTCCTGCT (REV).

### *In vitro* kinase assay

Full length Recombinant BTK (Invitrogen, PV3363), p53, and MDM2 were used in this study, the GST-tagged p53 and MDM2 were obtained by transfecting the appropriate pGEX vectors into BL21 competent and purifying the proteins produced with glutathione HiCap matrix beads (Qiagen #30900), as previously described [52]. 0.5 μg BTK and 0.5 μg p53 or MDM2 were added in a tube containing 25 μl of kinase buffer (12.5 mM Tris-HCl (pH7.5), 10 mM MgCl2, 1 mM EGTA, 0.5 mM Na3 VO4, 5 mM β-Glycerophosphate, 0.01% Triton X-100, 2 mM DTT, 200 μM ATP). Approximately 0.37 MBq of [32P] γ-ATP was added to each tube for 30 min at 30°C. Loading buffer was then added to each sample, and samples were boiled for 5 min and loaded into a 10% polyacrylamide gel. The gel was then dried using a Model v583 Gel drier (Biorad) for 1 hour and exposed to film in a Phosphor screen (GH Healthcare) overnight at room temperature. Phospho-image of dried gel was then developed using a Typhoon TRIO+ Variable mode manger scanner (GE Healthcare).

### *In vitro* ubiquitination assay

An *in vitro* ubiquitination assay was performed using an ubiquitination kit (Enzo Life Science, #BML-UW9920-0001). The GST-tagged p53 and MDM2 were obtained by transfecting the appropriate pGEX vectors into BL21 competent and purifying the proteins produced with glutathione HiCap matrix beads (Qiagen #30900), as previously described [52] and the reaction was prepared as follows: 10x ubiquitination buffer, 50 mM DTT, 0.1 M Mg-ATP, 20x E1 (His6-tagged recombinant human ubiquitin-activating enzyme), 10x E2 (His6-tagged recombinant human ubiquitin-conjugating enzyme UbcH7), 20x or 0.5 μg of E3 (GST-MDM2), 20x biotinylated ubiquitin, and 20x or 0.5 μg of target protein (GST-tagged full-length p53). This was incubated for 60 minutes at 37°C. The reaction was stopped by adding 10 μl 4x SDS-PAGE loading buffer and boiled for 5 minutes. 20 μl of the samples were run on SDS-PAGE gels and transferred to membranes. The amount and size of loaded protein was verified in one of the membranes using Instant Blue stain for 15 minutes at room temperature while rocking. The efficiency of ubiquitination was confirmed performing Western blotting on the second membrane.

### Immunofluorescence

Cells were processed and transfected following previously described protocols [53]. Cells were fixed using 4% paraformaldehyde, washed with PBS and permeabilised with 0.1% Triton X-100. Cells were then washed with PBS and blocked with 1% BSA. Coverslips were incubated overnight at 4°C with 1:100 anti-MDM2 (Abcam, #ab137413) or. The following day, coverslips were washed with PBS and incubated with 100 μl of 1:500 secondary anti-rabbit (Thermo Scientific, #84540) or anti-mouse antibody (Thermo Scientific, #35552) for 45 minutes in the dark. After incubation, coverslips were washed three times with PBS and stained with 4′,6-Diamidino-2-Phenylindole, Dihydrochloride (DAPI, Invitrogen) for 10 minutes. Slides were analysed using a Nokia TE300 semi-automatic microscope. The fluorescence intensity was measured using ImageJ analysis software (NIH, Bethesda, MD).

### Statistical analysis

All error bars represent the SD. Statistical significance (not significant, ns, *P* > 0.05; ^*^P ≤ 0.05; ^**^P ≤ 0.01; ^***^P ≤ 0.001; ^****^P ≤ 0.0001) was calculated using two-tailed unpaired *t* tests with Prism 6 (GraphPad) software.

## SUPPLEMENTARY MATERIALS FIGURE


