# Supplementary material for: BTK blocks the inhibitory effects of MDM2 on p53 activity
Source: Oncotarget. 2017 Nov 20;8(63):106639–47. doi: 10.18632/oncotarget.22543 (PMC5739762; doi:10.18632/oncotarget.22543)
Supplement: Supplementary file 1 [file oncotarget-08-106639-s001.pdf]

BTK blocks the inhibitory effects of MDM2 on p53 activity

SUPPLEMENTARY MATERIALS

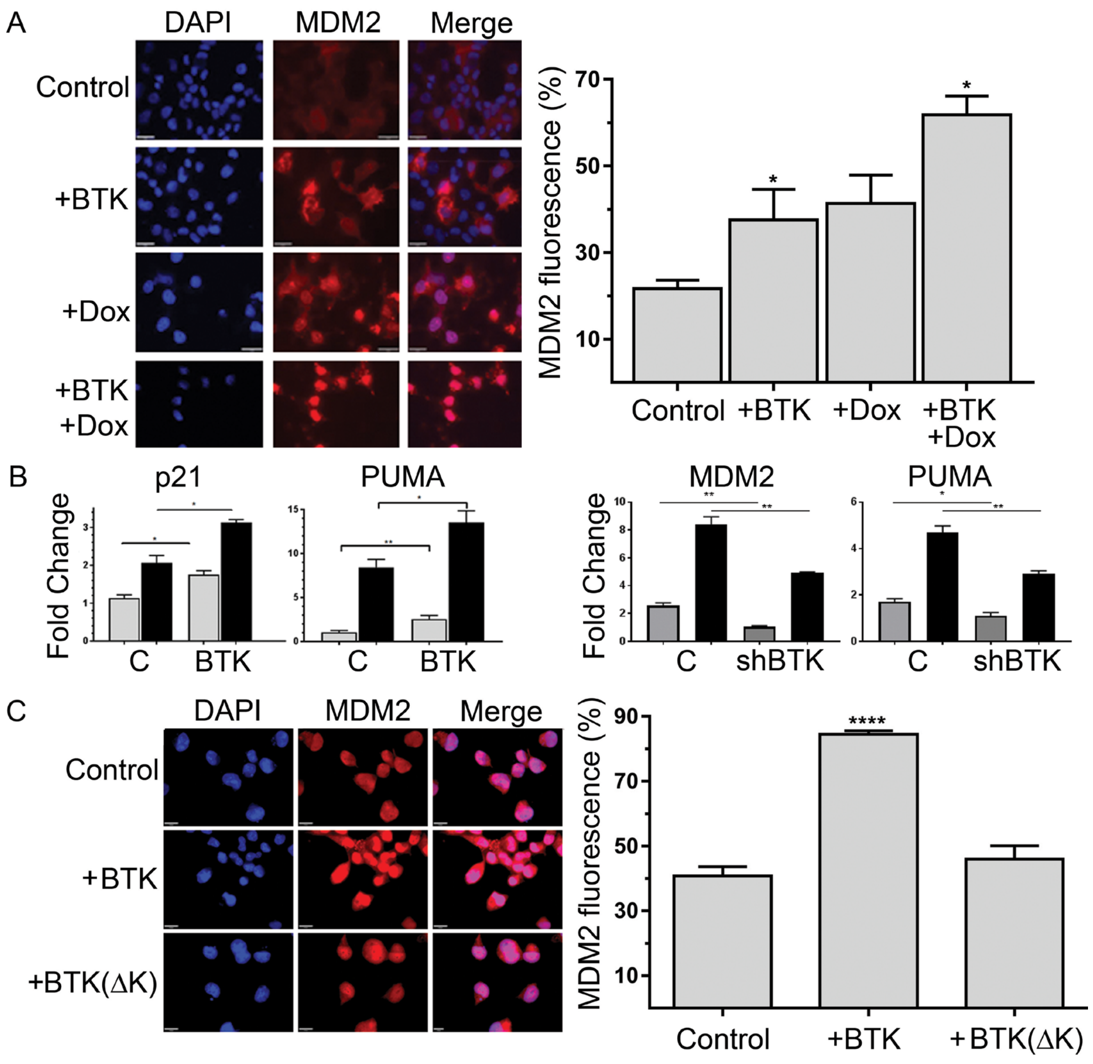

Supplementary Figure 1: (**A**) Representative immunofluorescence images of HCT116 cells transfected with and empty vector (Control) or BTK and treated with 1.5 µM doxorubicin for 24 h, showing expression of MDM2. DAPI is used to stain the DNA. Graphs shows percentage of fluorescence of MDM2 in relation to the total area, as measured by ImageJ analysis software. Results are the mean of three experiments and error bars show standard deviation. $^{*}P < 0.05$ (compared to Control). (**B**) ChIP perhormed in HCTT16 transfected with BTK (left two panels) or shRNA against BTK (right two panels), non-treated or treated with 1.5 µM doxorubicin (grey and black bars, respectively), showing binding of BTK to promoters of p53 target genes (MDM2, p21 and PUMA). Controls (C) are empty vector (left) or a shRNA against luciferase (right). $^{*}P \leq 0.05$; $^{**}P \leq 0.01$. (**C**) Representative immunofluorescence images of the same cells in Figure 2F. Graphs show percentage of fluorescence of BTK in relation to the total area, as measured by ImageJ analysis software. $^{****}P \leq 0.0001$ (compared to Control).
